# Retinal ganglion cell analysis in patients with sellar and suprasellar tumors with sagittal bending of the optic nerve

**DOI:** 10.1038/s41598-022-15381-6

**Published:** 2022-06-30

**Authors:** Yoichiro Shinohara, Daisuke Todokoro, Rei Yamaguchi, Masahiko Tosaka, Yuhei Yoshimoto, Hideo Akiyama

**Affiliations:** 1 Department of Ophthalmology, Gunma University Graduate School of Medicine, 3-39-15 Showa machi, Maebashi, Gunma 371–8511 Japan; 2 Department of Neurosurgery, Gunma University Graduate School of Medicine, Maebashi, Gunma Japan

**Keywords:** Eye diseases, Optic nerve diseases, Retinal diseases

## Abstract

The study investigated clinical features of sellar and suprasellar tumors with optic nerve bending. Twenty-five patients (13 men/12 women; age, 59.0 ± 12.9 years) with optic nerve bending in one eye who underwent tumor resection for sellar and suprasellar tumors were included. The other eye, without optic nerve bending, was the control. The pre- and postoperative best-corrected visual acuity (BCVA) and ganglion cell layer (GCL) + inner plexiform layer (IPL) thickness were studied retrospectively using optical coherence tomography. Preoperative BCVA in the eye with optic nerve bending was significantly poor and improved significantly after tumor resection. Eyes with optic nerve bending had significantly less GCL + IPL thickness on the temporal side than eyes without optic nerve bending. Preoperative GCL + IPL thickness of the entire macula was reduced in eyes with optic nerve bending and poor postoperative BCVA compared to those with good postoperative BCVA. There was no significant difference in GCL + IPL thickness of eyes with optic nerve bending before and after tumor resection. Optic nerve bending caused by sellar and suprasellar tumors resulted in visual impairment and decreased retinal ganglion cells. Eyes with optic nerve bending and severely reduced GCL + IPL thickness may have less BCVA improvement after tumor resection.

## Introduction

**Figure 1:**
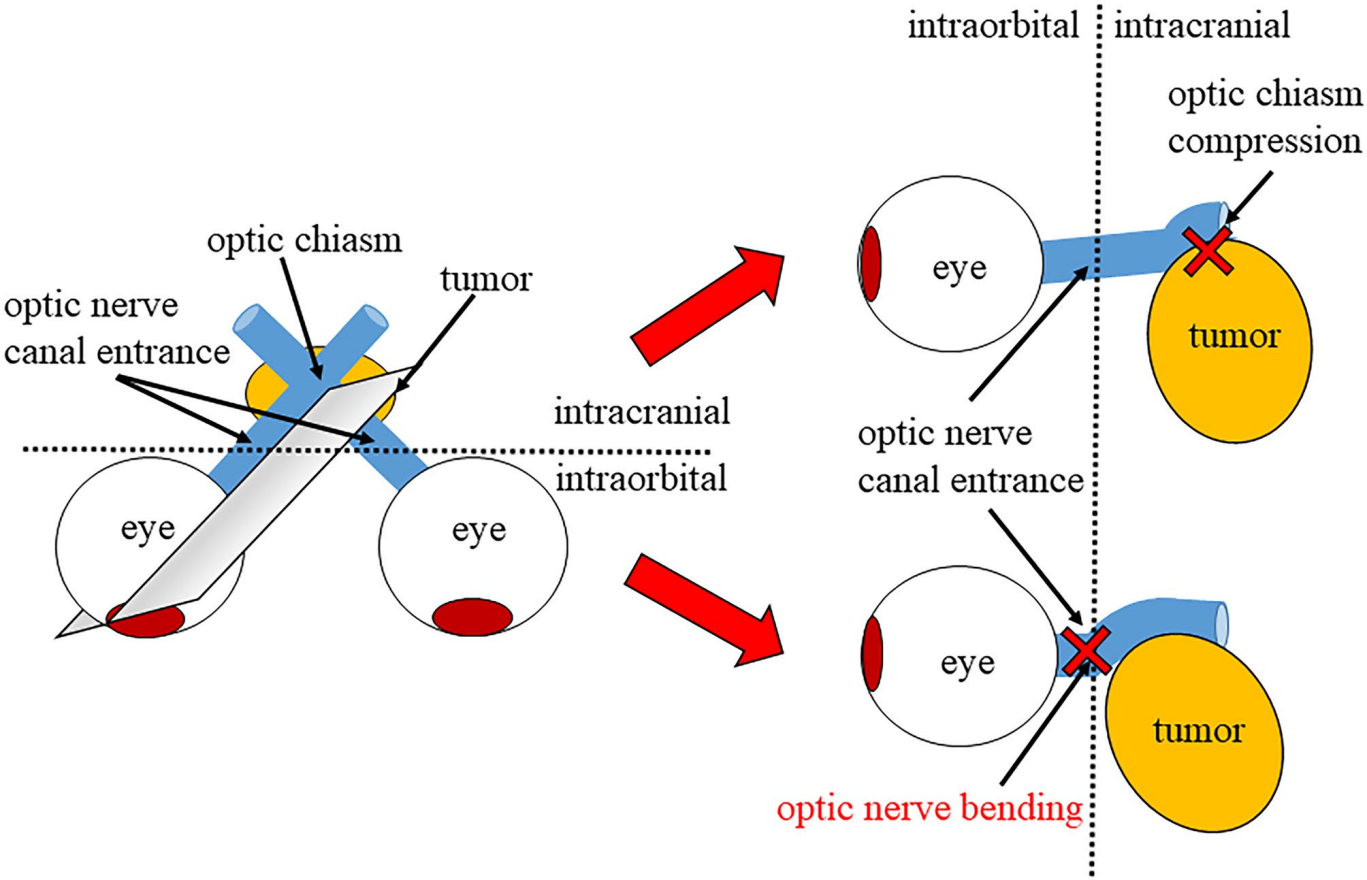
Schema from the eyeball to the optic chiasm in a patient with sellar and suprasellar tumor. The gray polygon in the left panel showed a cross-section of the path from the eyeball to the optic chiasm, as shown in the right panel. The upper right panel showed a schema of optic chiasm compression by the suprasellar and suprasellar tumor. The lower right panel showed a schema of optic nerve bending by the suprasellar and suprasellar tumor. The enlarged sellar and suprasellar tumor causes visual field defects due to optic chiasm compression. However, sellar and suprasellar tumors, with or without optic chiasm compression, sometimes bend the optic nerve at the optic canal’s entrance, leading to visual impairment.

Sellar and suprasellar tumors, including pituitary adenomas, show visual field defects, such as bilateral hemianopsia, due to the optic chiasm compression by the tumor^1^. Visual field impairment progresses gradually as the tumor grows, resulting in visual impairment^2,3^. However, even a relatively slight compression of the optic chiasm can cause visual impairment. We investigated the visual acuity impairment in the patients with sellar and suprasellar lesions using magnetic resonance (MR) T2 weighted imaging^4^. We also reported a new concept that the optic nerve sometimes bends at the entrance of the optic canal from the intracranial subarachnoid space. Further, a combination of preoperative optic nerve bending and optic chiasm compression is associated with visual impairment (Fig. 1)^4^. The effect of optic nerve bending caused by sellar and suprasellar tumors on the retina remains unclear. Optical coherence tomography (OCT) can measure ganglion cell layer (GCL) + inner plexiform layer (IPL) thickness in the macula with high reliability. Previous reports showed the usefulness of macular GCL + IPL parameters in discriminating early glaucoma from normal eyes^5^. Morphological changes in retinal ganglion cells might also be observed using OCT in patients with sellar and suprasellar tumors with optic nerve bending. Here, we investigated the OCT findings of patients with sellar and suprasellar tumors with optic nerve bending before and after tumor resection.

## Results

### Demographic characteristics and visual acuity

**Figure 2:**
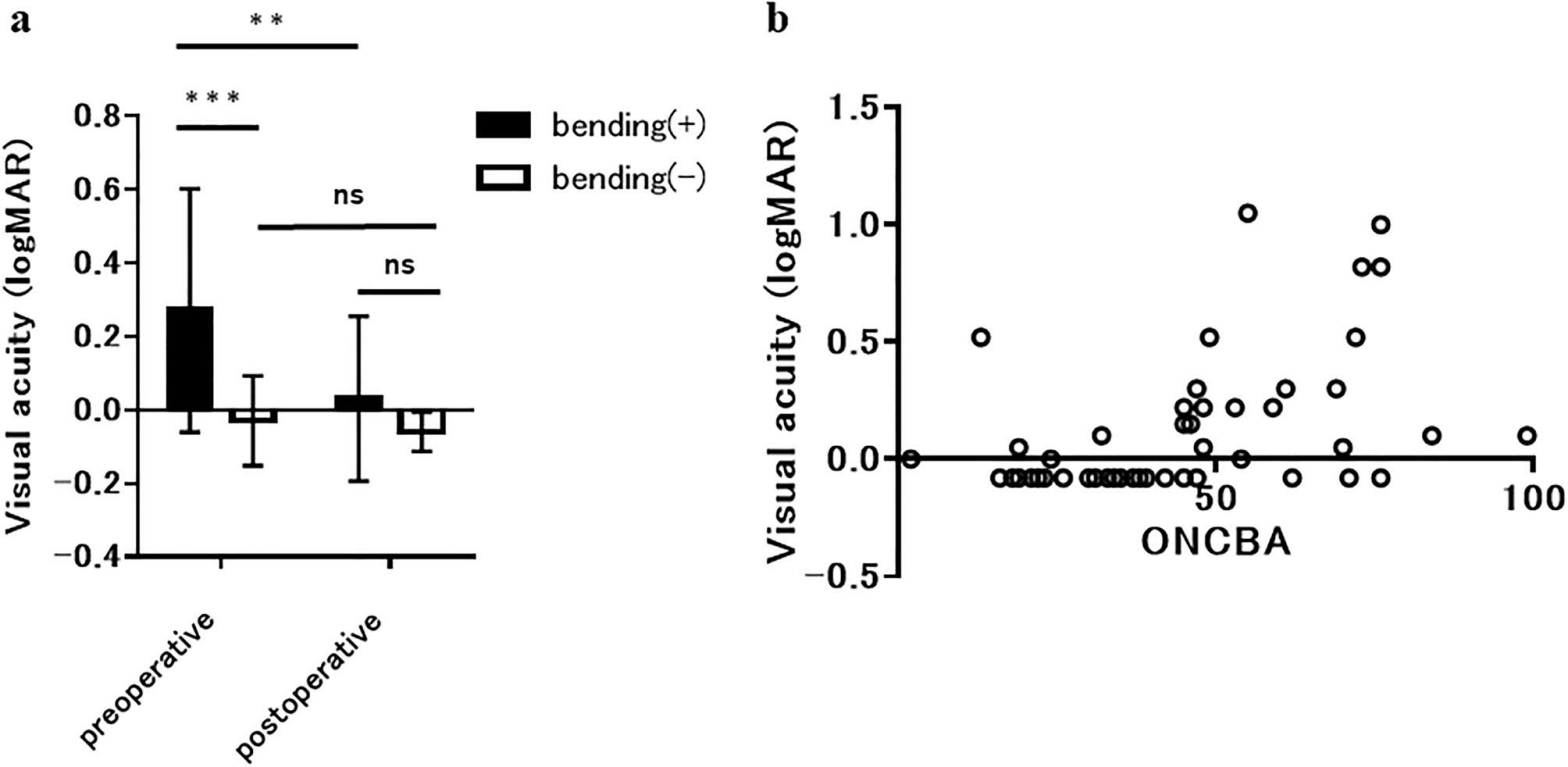
Assessment of best-corrected visual acuity (BCVA) in patients with the sellar and suprasellar tumor. (**a**) Comparison of BCVA between eyes with and without optic nerve bending before and after surgery. The preoperative BCVA of eyes with optic nerve bending was significantly lower than those without optic nerve bending (**p < 0.001). BCVA was significantly improved after tumor resection in eyes with optic nerve bending (**p < 0.001). There was no significant difference between the pre- and postoperative BCVA of eyes without optic nerve bending (p = 0.40). (**b**) Graphs showing the correlation between the ONCBA and preoperative BCVA in 50 eyes of 25 patients. The ONCBA had a positive correlation with preoperative BCVA (r = 0.4986, p = 0.0002).

Table 1 shows patients' demographic and clinical characteristics with sellar and suprasellar tumors. Regarding the sellar and suprasellar tumors, 15, 4, 3, and 3 patients had pituitary adenoma, craniopharyngioma, Rathke’s cleft cyst, and meningioma, respectively. Patients with the sellar and suprasellar tumors included 13 men (52.0%) and 12 women (48.0%). Their average age was 59.0 ± 12.9 years. The mean optic nerve–canal bending angles (ONCBAs) were 56.7 ± 11.0° in bending eyes and 27.2 ± 8.4° in non-bending eyes (p < 0.001). Tables 2 and 3 show ONCBA and ophthalmologic parameters of all patients. Preoperative and postoperative best-corrected visual acuities (BCVAs) (logMAR) were 0.27 ± 0.33 and − 0.03 ± 0.12 and 0.03 ± 0.23 and − 0.06 ± 0.05 in bending and non-bending eyes, respectively (Fig. 2a). Preoperative BCVA of the bending eyes was significantly lower than that of the non-bending eyes (p < 0.001). Postoperative BCVA was significantly better than preoperative BCVA in the bending eyes (p < 0.01) (Fig. 2a). No significant differences were found between the preoperative and postoperative BCVA in non-bending eyes. In addition, Spearman’s correlation (r = 0.4986, p = 0.0002) shows that ONCBA was positively correlated with preoperative BCVA in 50 eyes of 25 patients (Fig. 2b). Postoperative BCVA and GCL + IPL thickness in all sectors did not correlate with ONCBA.

**Table 1:** Systemic characteristics of patients with sellar and suprasellar tumors.

	Subject (n = 25)	
	Optic nerve bending (+)	Optic nerve bending (−)
Number of eyes (n)	25	25
Age (years)	59.0 ± 12.9	
Male:female (n)	13:12	
**Pathology (n)**		
Pituitary adenoma	15	
Craniopharyngioma	4	
Rathke’s cleft cyst	3	
Meningioma	3	
Right eye (n)	14	11
Left eye (n)	11	14
ONCBA (°)	56.7 ± 11.0	27.2 ± 8.4

*ONCBA* optic nerve–canal bending angle.

**Table 2:** Angle of bending and ophthalmologic parameters of patients with optic nerve bending.

No.	ONCBA	Pre-op							Post-op						
		GCIPL (µm)							GCIPL (µm)						
		BCVA	S	SN	IN	I	IT	ST	BCVA	S	SN	IN	I	IT	ST
1	46	0.15	65	63	62	67	73	72	0.05	66	63	61	66	73	72
2	76	1.00	67	67	65	67	73	69	− 0.08	63	62	60	63	71	66
3	54	0.00	78	77	69	70	80	79	− 0.08	78	76	69	69	78	79
4	76	0.82	63	64	65	69	75	69	− 0.08	62	62	62	67	74	66
5	59	0.22	79	82	77	76	85	82	− 0.08	79	82	74	74	84	81
6	99	0.10	61	61	56	55	59	56	0.05	62	61	56	56	58	58
7	48	0.05	84	83	80	80	83	83	− 0.08	85	83	79	78	82	81
8	72	0.52	60	59	56	58	73	65	− 0.08	62	60	55	59	70	65
9	45	0.22	67	67	65	70	78	73	0.00	65	65	62	73	79	73
10	70	0.05	67	64	61	70	79	73	− 0.08	66	63	61	68	79	72
11	53	0.22	83	87	84	78	78	79	− 0.08	82	86	82	74	75	76
12	84	0.10	62	54	55	66	83	78	− 0.08	61	53	54	64	82	77
13	73	0.82	48	51	48	54	63	53	0.70	49	49	47	53	60	50
14	47	− 0.08	74	68	64	72	84	83	− 0.08	75	68	63	71	83	82
15	55	1.05	23	30	27	23	23	26	0.70	24	23	24	21	23	22
16	71	− 0.08	59	59	57	64	78	68	− 0.08	56	56	53	62	75	66
17	76	− 0.08	77	79	75	74	81	77	− 0.08	78	79	75	75	83	79
18	45	− 0.08	60	46	44	59	74	72	− 0.08	61	47	46	60	75	73
19	49	0.52	73	72	71	75	80	75	− 0.08	73	72	71	75	80	75
20	62	− 0.08	71	79	73	65	63	67	− 0.08	66	74	76	71	62	67
21	69	0.30	68	64	58	63	75	73	0.40	66	63	58	64	75	71
22	47	0.30	79	74	70	74	83	78	− 0.08	80	75	70	75	83	79
23	45	0.15	57	53	52	62	77	71	0.15	56	53	51	59	75	72
24	48	0.22	48	46	46	51	57	58	0.15	49	46	45	51	57	58
25	61	0.30	68	70	68	34	65	68	− 0.08	67	68	65	67	71	68

*ONCBA* optic nerve–canal bending angle, *BCVA* best-corrected visual acuity(logMAR).

**Table 3:** Angle of bending and ophthalmologic parameters of patients without optic nerve bending.

No.	ONCBA	Pre-op							Post-op						
		GCIPL(µm)							GCIPL(µm)						
		BCVA	S	SN	IN	I	IT	ST	BCVA	S	SN	IN	I	IT	ST
1	32	0.10	72	69	60	64	77	75	0.10	66	68	60	68	74	70
2	23	− 0.08	66	64	62	67	75	67	− 0.08	65	62	57	66	76	67
3	34	− 0.08	79	81	77	74	82	78	− 0.08	79	79	74	73	80	82
4	31	− 0.08	73	70	68	72	76	80	− 0.08	73	70	67	71	76	78
5	22	− 0.08	77	77	72	75	87	80	− 0.08	79	76	78	73	84	83
6	2	0.00	66	59	53	59	68	68	0.00	66	61	54	57	69	68
7	33	− 0.08	86	83	79	85	92	89	− 0.08	86	83	79	83	91	88
8	39	− 0.08	58	54	51	56	70	68	− 0.08	59	54	52	56	68	68
9	19	0.05	74	68	61	63	73	74	− 0.08	72	68	63	65	75	72
10	21	− 0.08	72	58	58	79	91	88	− 0.08	73	57	57	78	90	87
11	19	− 0.08	87	88	81	82	88	81	− 0.08	83	85	79	81	88	80
12	24	0.00	66	62	58	71	85	78	− 0.08	64	61	57	68	83	78
13	39	− 0.08	60	53	50	57	68	70	− 0.08	62	53	51	56	67	70
14	30	− 0.08	79	73	70	70	80	82	− 0.08	78	72	70	72	79	80
15	38	− 0.08	66	61	57	63	71	69	− 0.08	65	61	56	62	70	68
16	37	− 0.08	79	73	68	77	85	83	0.05	77	70	64	75	82	80
17	42	− 0.08	81	83	79	79	79	77	− 0.08	81	82	81	80	80	77
18	18	− 0.08	51	47	46	59	72	59	− 0.08	52	47	46	59	72	62
19	26	− 0.08	70	69	64	75	84	76	− 0.08	70	69	64	75	84	76
20	16	− 0.08	70	77	77	75	77	72	− 0.08	71	78	76	71	75	72
21	42	− 0.08	71	68	64	72	125	85	− 0.08	71	66	64	71	130	86
22	35	− 0.08	83	77	71	75	84	83	− 0.08	82	77	71	74	82	83
23	13	0.52	50	50	48	57	69	58	0.10	52	50	49	55	66	57
24	24	0.00	67	71	65	65	74	71	− 0.08	68	70	65	65	74	72
25	37	− 0.08	82	85	82	80	82	84	− 0.08	82	85	81	80	83	85

*ONCBA* optic nerve–canal bending angle, *BCVA* best-corrected visual acuity(logMAR).

All 25 patients with optic nerve bending were classified by age into groups of 30–40 s (n = 6), 50 s (n = 7), 60 s (n = 5), and 70 s (n = 7). Postoperative BCVAs (logMAR) were − 0.08 ± 0.00 (30–40 s),0.01 ± 0.17 (50 s), 0.08 ± 0.31 (60 s) and 0.13 ± 0.25 (70 s), respectively. Postoperative BCVA showed no significant differences among these 4 groups (all p > 0.05, a one-way analysis of variance followed by Tukey’s post hoc test).

### Assessment of GCL + IPL using OCT

**Figure 3:**
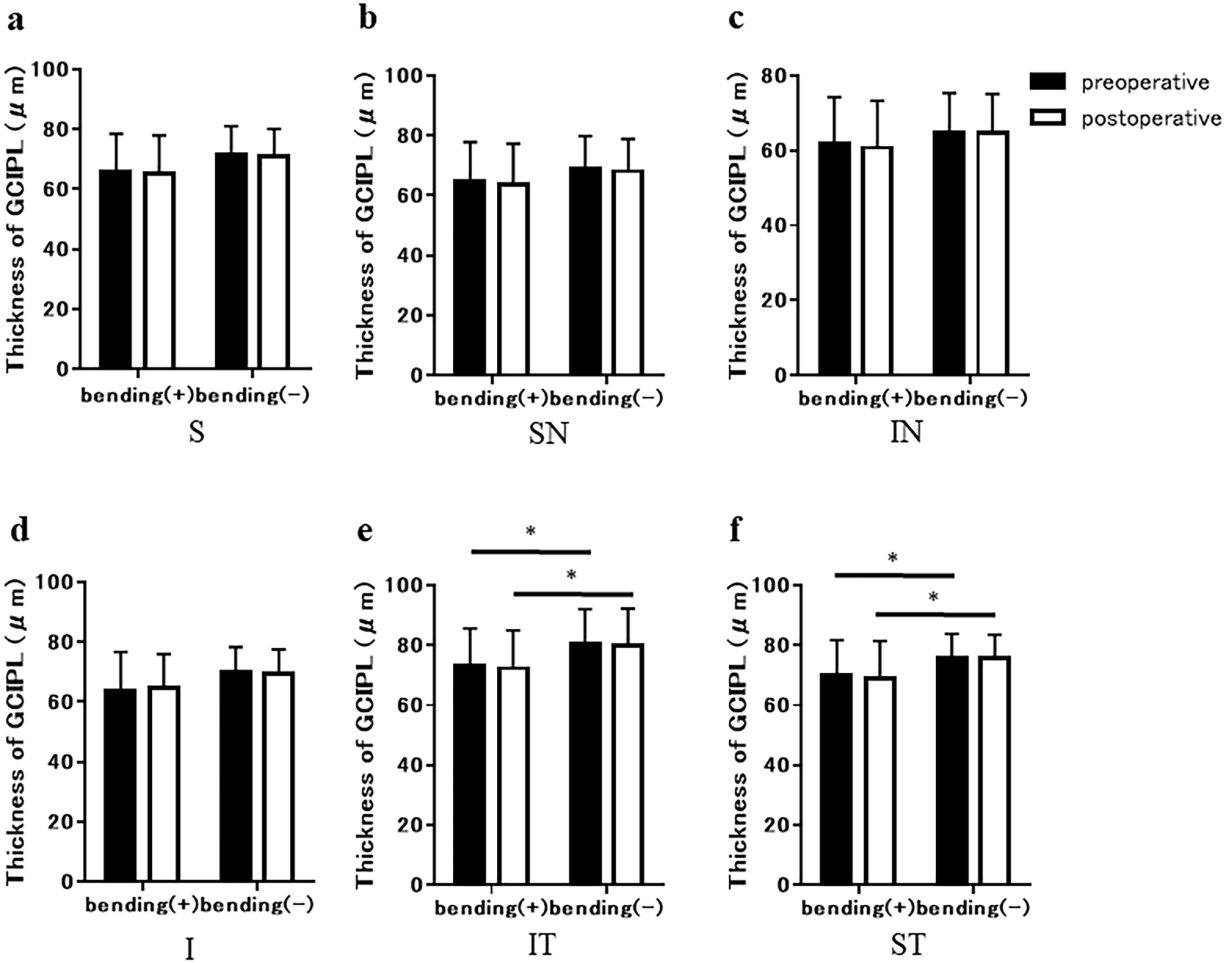
Comparison of ganglion cell layer (GCL) + inner plexiform layer (IPL) thickness between preoperatively (black bar) and postoperatively (white bar) in eyes with and without optic nerve bending. Graphs showed the superior (S) (**a**), superior nasal (SN) (**b**), inferior nasal (IN) (**c**), inferior (I) (**d**), inferior temporal (IT) (**e**), and superior temporal (ST) (**f**) sectors of the macular, respectively. There was no significant difference in pre- and postoperative GCL + IPL thickness in all sectors (all p > 0.05). Both preoperatively and postoperatively, GCL + IPL thickness in the inferior temporal (IT) and superior temporal (ST) sectors was significantly lesser in eyes with optic nerve bending than in eyes without optic nerve bending (both *p < 0.05).

The preoperative GCL + IPL thicknesses in bending and non-bending eyes were 65.6 ± 12.8 µm and 71.4 ± 9.7 µm in the superior sector, 64.8 ± 13.1 µm and 68.8 ± 11.1 µm in the superior nasal sector, 61.9 ± 12.4 µm and 64.8 ± 10.6 µm in the inferior nasal sector, 63.8 ± 12.9 µm and 70.0 ± 8.4 µm in the inferior sector, 72.9 ± 12.8 µm and 80.6 ± 11.5 µm in the inferior temporal sector, and 69.9 ± 11.8 µm and 75.8 ± 8.0 µm in the superior temporal sector, respectively. The postoperative GCL + IPL thicknesses in the bending and non-bending eyes were 65.2 ± 12.7 µm and 71.0 ± 9.1 µm in the superior sector, 63.6 ± 13.7 µm and 68.2 ± 10.7 µm in the superior nasal sector, 60.8 ± 12.6 µm and 64.6 ± 10.5 µm in the inferior nasal sector, 64.6 ± 11.4 µm and 68.4 ± 8.2 µm in the inferior sector, 72.3 ± 12.7 µm and 79.9 ± 12.3 µm in the inferior temporal sector, and 69.1 ± 12.4 µm and 75.6 ± 8.0 µm in the superior temporal sector, respectively (Fig. 3a–f). There was no significant difference in GCL + IPL thickness before and after surgery in all sectors (all p > 0.05). In both bending and non-bending eyes, the GCL + IPL thickness was lesser in the nasal sectors than in the temporal sectors, both before and after surgery. Notably, the GCL + IPL thickness in the superior temporal and inferior temporal sectors was significantly lesser in the bending eyes than in the non-bending eyes, both before and after surgery (p < 0.05) (Fig. 3e,f).

**Figure 4:**
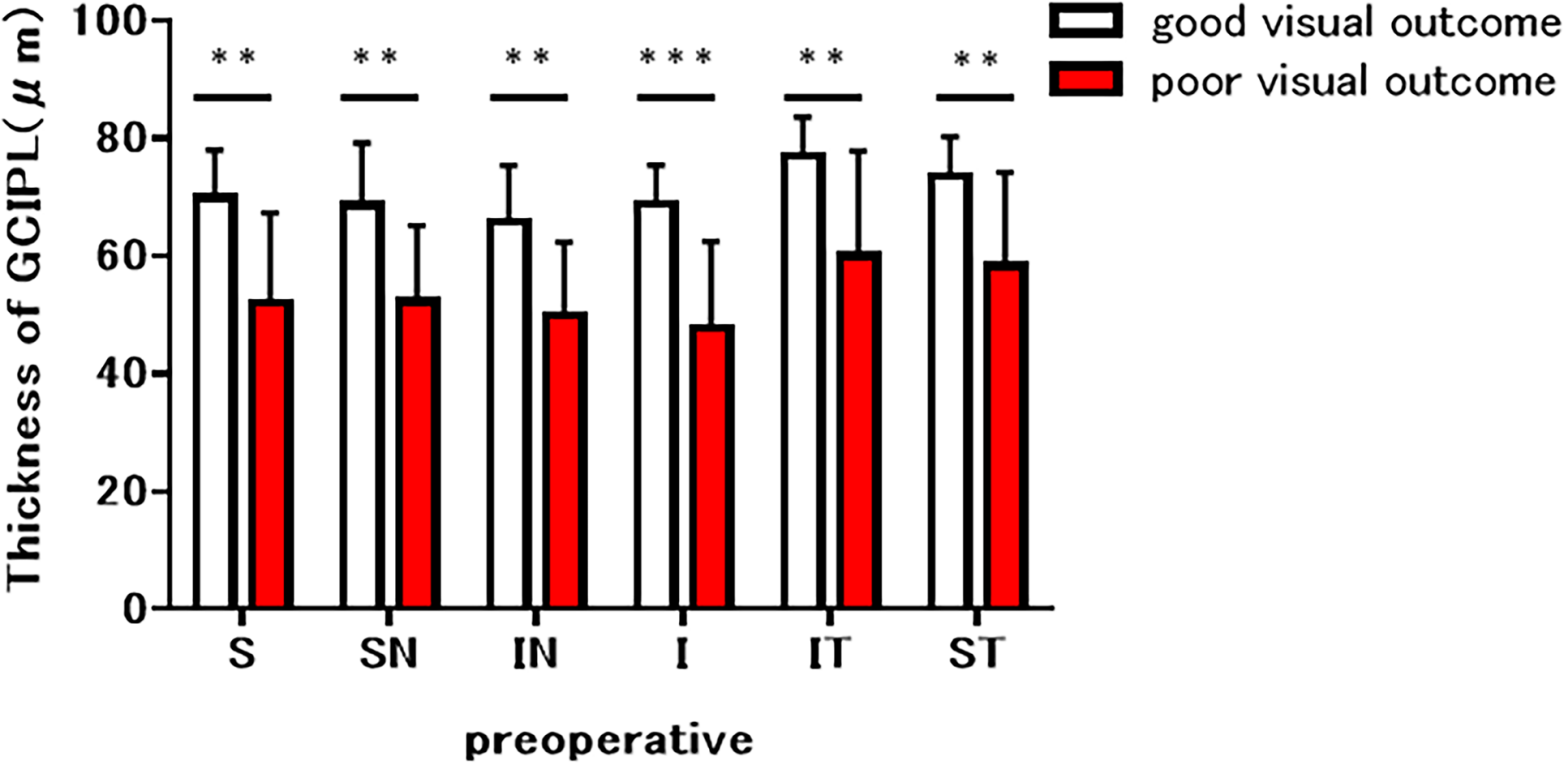
Comparison of preoperative ganglion cell layer (GCL) + inner plexiform layer (IPL) thickness between eyes with and without postoperative visual disturbance in eyes with optic nerve bending. Graphs showed the superior (S), superior nasal (SN), inferior nasal (IN), inferior (I), inferior temporal (IT), and superior temporal (ST) sectors of the macular, respectively. Preoperative GCL + IPL thickness was lesser in eyes with postoperative visual disturbance than in eyes with postoperative non-visual disturbance in all sectors (***p < 0.001, inferior sector; **p < 0.01, other 5 sectors).

**Figure 5:**
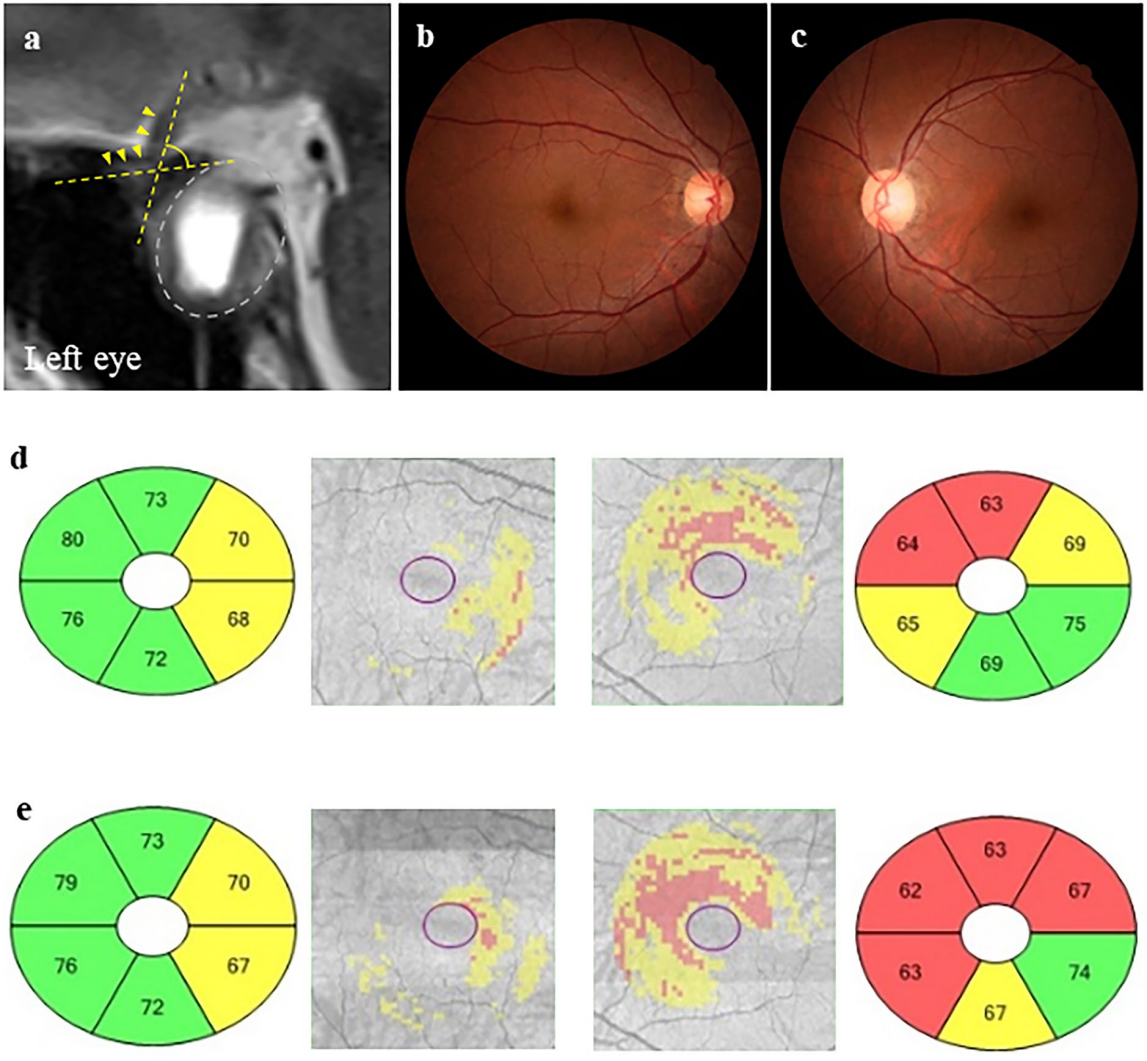
Representative clinical findings of a case with good visual outcome (case 1). A 63-year-old woman with pituitary adenoma with optic nerve bending in the left eye. Preoperative and postoperative best-corrected visual acuities (BCVAs) were − 0.08 and − 0.08 in the right eye and 0.82 and − 0.08 in the left eye logarithm of the minimum angle of resolution units, respectively. (**a**) Preoperative sagittal T2-weighted magnetic resonance images. The optic nerve–canal bending angle (ONCBA) was formed by the optic nerve (nerve indicated by yellow arrowheads) in the optic canal and the optic nerve in the intracranial subarachnoid space at the optic canal’s exit (angle indicated by yellow dotted lines). White dotted lines indicate a tumor. The ONCBAs of this case were 31° in the right eye and 76° in the left eye. Therefore, the left eye is the one with optic nerve bending. (**b**, **c**) Color fundus photograph showing the normal appearance at preoperative visit (B, right eye; C, left eye). (**d**, **e**) Ganglion cell layer (GCL) + inner plexiform layer (IPL) deviation map showing the expansion from the parafovea to the periphery of the red area indicates the decrease of GCL + IPL thickness and outside normal limits. GCL + IPL deviation map and sector graph of the right eye (left 2 panels) and left eye (right 2 panels) show a decrease in the macular GCL + IPL thickness, especially from nasal to superior sectors in the left eye. A slight decrease in postoperative GCL + IPL thickness is observed in the left eye (**d**, preoperative; **e**, postoperative visit).

**Figure 6:**
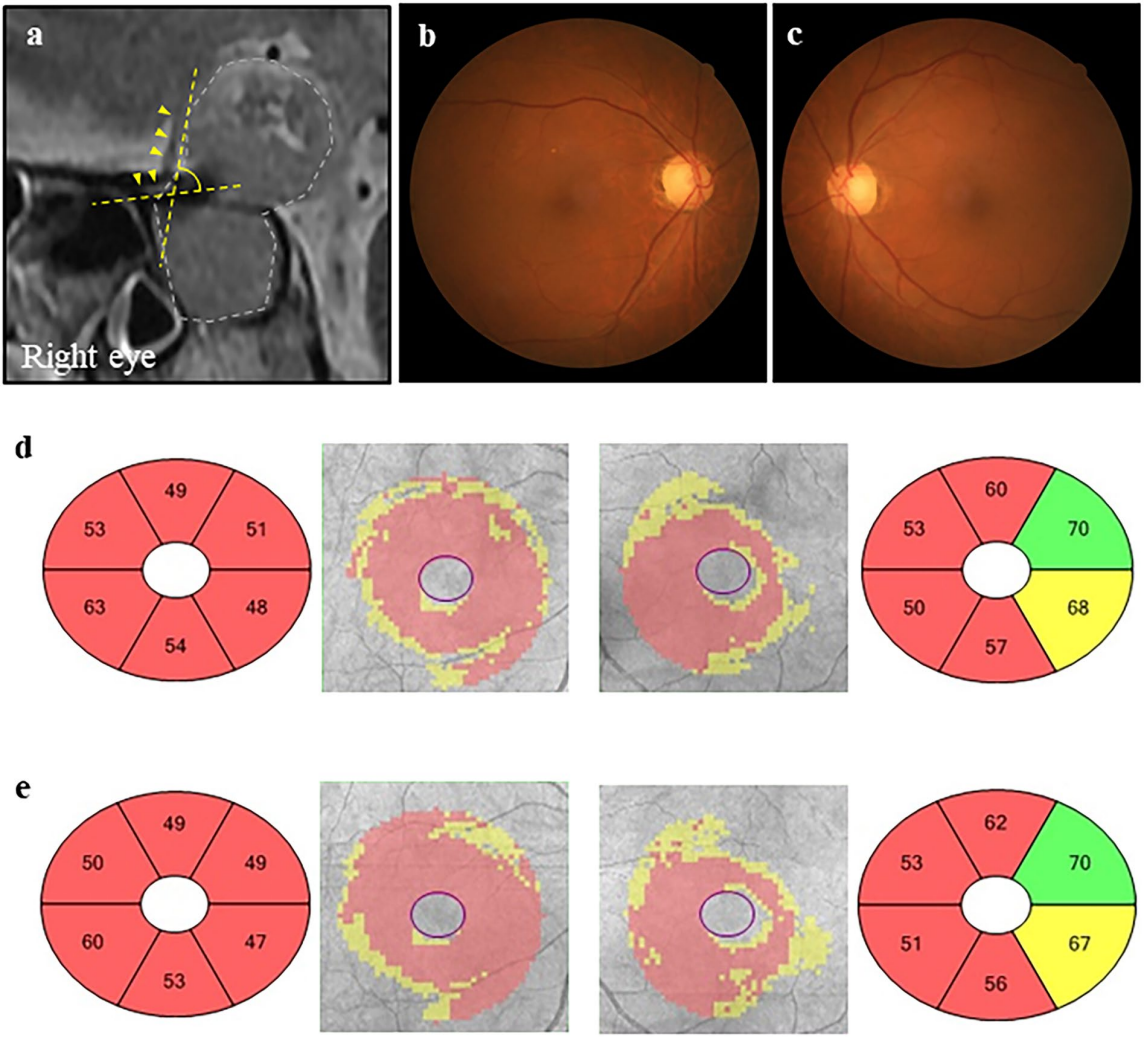
Representative clinical findings of a case with poor visual outcome (case 2). A 74-year-old man with pituitary adenoma with optic nerve bending in the right eye. The preoperative and postoperative best-corrected visual acuities (BCVAs) were 0.82 and 0.10 in the right eye and 0.70 and − 0.08 in the left eye logarithm of the minimum angle of resolution units, respectively. (**a**) Preoperative sagittal T2-weighted magnetic resonance images. The angle indicated by yellow dotted lines is the optic nerve–canal bending angle (ONCBA); yellow arrowheads indicate optic nerve, and white dotted lines indicate a tumor. The ONCBAs of this case were 73° in the right eye and 39° in the left eye. Therefore, the right eye is the one with optic nerve bending. (**b**, **c**) Color fundus photograph showed normal appearance at preoperative visit (B, right eye; C, left eye). (**d**, **e**) Ganglion cell layer (GCL) + inner plexiform layer (IPL) deviation map and sector graph of the right eye (left 2 panels) and left eye (right 2 panels) (**d**, preoperative; **e**, postoperative visit). A decrease in preoperative GCL + IPL thickness of the right eye is observed in all sectors. A decrease in preoperative GCL + IPL thickness of the left eye is observed in the predominantly nasal region. Postoperative GCL + IPL thickness in almost all sectors was more unremarkable than preoperative GCL + IPL thickness in both eyes.

In bending eyes, we defined 19 eyes with postoperative BCVA (logMAR) of 0 or better as good visual outcome and 6 eyes with postoperative BCVA (logMAR) of less than 0 as poor visual outcome. The preoperative GCL + IPL thicknesses in the good visual outcome eyes and the poor visual outcome eyes were 69.9 ± 8.1 µm and 52.0 ± 15.3 µm in the superior sector, 68.7 ± 10.5 µm and 52.3 ± 12.8 µm in the superior nasal sector, 65.7 ± 9.6 µm and 49.8 ± 12.5 µm in the inferior nasal sector, 68.9 ± 6.6 µm and 47.8 ± 14.6 µm in the inferior sector, 76.9 ± 6.7 µm and 60.0 ± 17.9 µm in the inferior temporal sector, and 73.6 ± 6.7 µm and 58.2 ± 16.0 µm in the superior temporal sector, respectively (Fig. 4). The preoperative GCL + IPL thickness in eyes with poor visual outcome was significantly lesser than that in eyes with good visual outcomes in all sectors (p < 0.001, inferior sector; p < 0.01, other 5 sectors). Representative images of a patient with pituitary adenoma with optic nerve bending with good and poor visual outcomes are shown in Figs. 5 and 6.

## Discussion

We retrospectively investigated the clinical features, including retinal ganglion cells (RGCs) analysis, of sellar and suprasellar tumors with sagittal bending of the optic nerve and compared them with those of non-bending optic nerve controls. Eyes with optic nerve bending due to sellar and suprasellar tumors had worse visual acuity and reduced GCL + IPL thickness in the temporal sectors, as measured by OCT, than eyes without optic nerve bending. In addition, eyes with optic nerve bending showed rapid improvement in visual acuity after tumor resection. Furthermore, in six eyes with poor visual outcome, the preoperative GCL + IPL thickness was significantly lesser than that in 19 eyes with good visual outcome.

Yamaguchi et al. measured the sagittal angle of the optic nerve at the entrance of the optic canal using MR imaging in patients with sellar and suprasellar tumors and reported a new concept that sellar and suprasellar tumors cause not only optic chiasm compression but also optic nerve bending, resulting in visual impairment^4^. ONCBA basically affects ipsilateral vision^4^. Moreover, ipsilateral ONCBA is anatomically unrelated to contralateral visual dysfunction. However, when the ONCBA is large, the tumor is often large; therefore, the visual field defect due to chiasma compression may occur bilaterally. In addition, if the tumor is larger, the ONCBA on the contralateral side may be large. In this study, eyes with optic nerve bending had preoperative visual impairment, whereas eyes without optic nerve bending had good preoperative visual acuity (Fig. 2a). The mechanism of visual impairment due to optic nerve bending caused by sellar and suprasellar tumors remains unknown. The optic nerve at the entrance of the optic canal receives blood flow mainly from the superior pituitary artery, with little blood flow from the ophthalmic artery, which is prone to ischemia. The optic chiasm is rich in blood flow, supplied by branches from the internal carotid artery, anterior cerebral artery, and anterior communicating artery^6,7^. Hence, the optic nerve bending may be more likely to cause visual impairment due to ischemia than optic chiasm compression because the optic nerve at the optic canal’s entrance has less blood flow than the optic chiasm.

The nasal GCL + IPL thickness is reduced in pituitary adenomas compared to normal subjects because tumor-induced optic chiasm compression damages the crossed fibers and retrogradely damages retinal ganglion cells^8^. In the current study, the GCL + IPL thickness was also lesser in the nasal sectors of sellar and suprasellar tumors with and without optic nerve bending. Notably, the GCL + IPL thickness in the temporal sectors was significantly lesser in bending eyes than in non-bending eyes. (Fig. 3e,f). Tumor-induced optic nerve bending at the entrance of the optic canal causes compression of the bony margin of the optic canal and stretching of the local optic nerve. Local compression of the optic nerve by bending in the narrow space of the optic canal’s entrance may cause more dysfunction of the entire optic nerve cord than local compression in the relatively wide space of the optic chiasm, which may affect not only the nasal GCL + IPL but also the temporal GCL + IPL.

Transsphenoidal surgery is an effective and safe treatment for most patients with pituitary adenomas and is expected to improve visual function^9^. Eyes with sellar and suprasellar tumors with optic nerve bending had severe visual impairment, but visual acuity improved with tumor resection (Fig. 2a). In contrast, eyes with optic nerve bending that showed little improvement in postoperative BCVA showed a significant decrease in preoperative GCL + IPL thickness (Fig. 4). Several factors have been previously investigated to predict visual recovery after optic chiasm decompression surgery. Retinal nerve fiber layer (RNFL) thinning, which reflects the loss of ganglion cell axons, is a predictor of poor visual recovery after surgery due to the optic chiasm compression and permanent denervation of the optic radiations and visual cortex^10–12^. Previous reports have shown that eyes with a normal RNFL have improved visual fields postoperatively compared to eyes with a thin RNFL^13^. In a representative case of good visual outcome group (case 1), the preoperative OCT of the optic nerve bending eye showed only a mild decrease in GCL + IPL in the predominantly superior nasal sector, and the postoperative BCVA improved (Fig. 5). In contrast, in a representative case of poor visual outcome group (case 2), preoperative OCT of the optic nerve bending eye revealed severe GCL + IPL reduction in all sectors, and postoperative BCVA did not improve (Fig. 6). Based on this result, the preoperative GCL + IPL thickness measured by OCT may predict the prognosis of postoperative visual function in sellar and suprasellar tumors with optic nerve bending. Furthermore, retinal ganglion cell death may progress faster with optic nerve bending than with optic chiasm compression. Prolonged visual symptoms in pituitary adenomas have been reported to decrease the improvement in visual function after tumor resection^13^. In optic chiasm compression and optic nerve bending, the optic nerve compression period may be associated with a decrease in RGCs. This study did not examine the time between the onset of visual impairment and ophthalmologic evaluation. The duration since optic nerve bending onset may affect visual impairment; hence, further studies are needed in the future. A previous report indicated that measuring RGCs may identify nerve fiber damage before RNFL in homonymous hemianopia^14^. Although not examined in this study, sellar and suprasellar tumors with optic nerve bending may also show changes in RNFL following RGCs. The limitations of our study include its retrospective nature, single-center design, and small sample size. Further research needs to include a large multi-center study.

In conclusion, sellar and suprasellar tumors with optic nerve bending cause thinning of the RGCs on the nasal and temporal sides. Eyes with optic nerve bending and severe retinal ganglion cell thinning had poor visual acuity even after tumor resection, and preoperative GCL + IPL thickness may be a prognostic factor for postoperative visual acuity.

## Methods

### Subjects and measurement of clinical examinations

All experiments followed the tenets of the Declaration of Helsinki and were approved by the Institutional Review Board of the Gunma University Graduate School of Medicine (HS2021-097). Informed consent was obtained from all individual participants in the present study. We retrospectively studied 25 patients with visual impairment due to sellar and suprasellar tumors who underwent endoscopic transsphenoidal tumor resection at Gunma University Hospital from June 2015 to July 2021 and had optic nerve bending in only one eye. The other eye, without optic nerve bending, was used as the control. MR imaging of the sellar and suprasellar lesions in all 25 patients with a 1.5 T or 3 T MR imaging system was performed. The presence of optic nerve bending was determined by measuring the sagittal ONCBA on MR images before tumor resection, as previously reported^4^. Briefly, the ONCBA is the angle obtained by neurosurgeons measuring the extent of this bending on sagittal MR images formed by the optic nerve in the optic canal and the optic nerve in the intracranial subarachnoid space at the entrance of the optic canal. Each neurosurgeon specializing in pituitary tumor MR reading and surgery (R.Y. and M.T.) made evaluations, and any disagreements regarding conclusions were resolved by consensus. Optic nerve bending (large ONCBA) was defined as ONCBA ≥ 45°, and non-optic nerve bending (moderate ONCBA) was defined as ONCBA < 45°, as previously reported^4^. The exclusion criteria were as follows: (1) patients with a history of glaucoma or evident glaucomatous optic neuropathy; (2) high myopia (refractive error less than − 6 diopters); (3) retinal diseases, including epiretinal membrane and macular edema; (4) severe cataract, and (5) unclear optic nerve on MR imaging.

**Figure 7:**
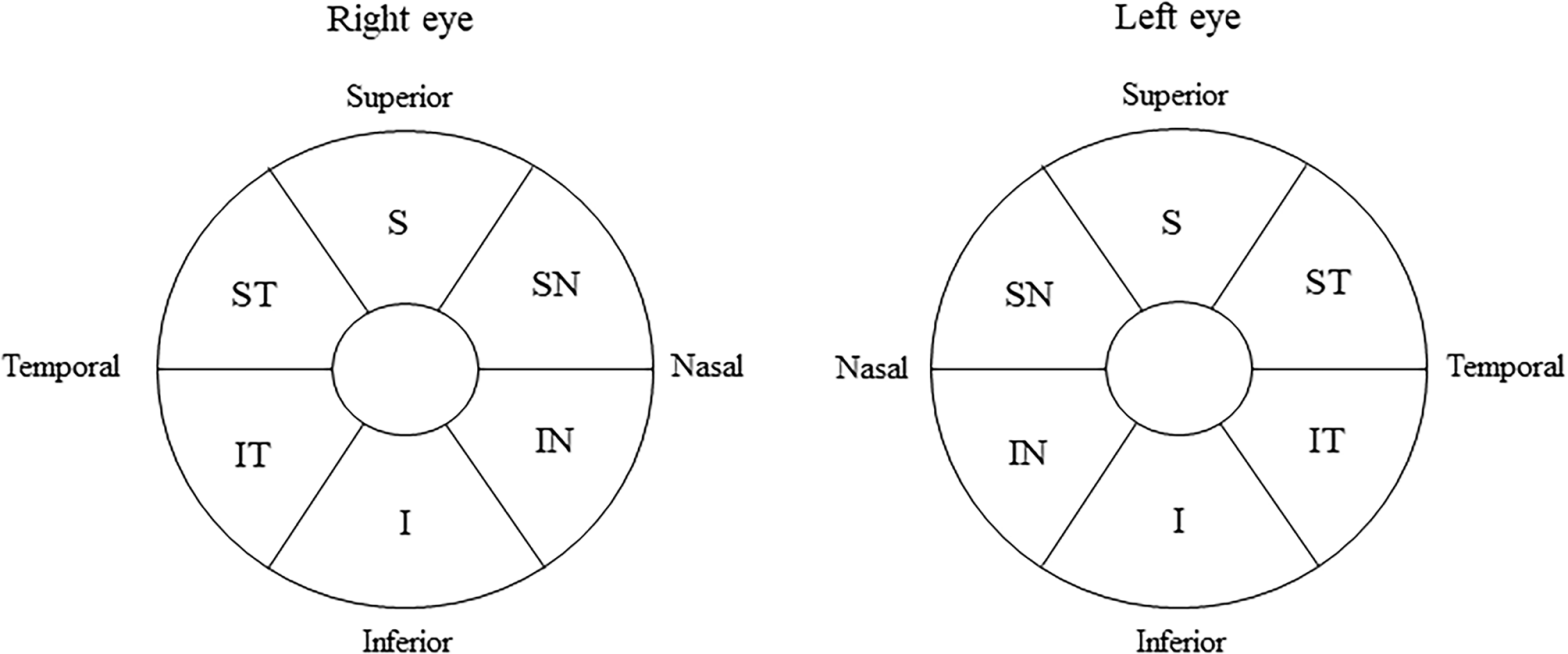
Ganglion cell layer + inner plexiform layer sector map of both eyes using optical coherence tomography. The macular region was divided into six sectors: superior (S), superior nasal (SN), inferior nasal (IN), inferior (I), inferior temporal (IT), and superior temporal sectors (ST).

All patients underwent ophthalmologic examinations, including best-corrected visual acuity (BCVA), intraocular pressure assessment, refraction, slit-lamp biomicroscopy, fundus examination, and GCL + IPL thickness measurement, using Cirrus high definition-OCT (Carl Zeiss Meditec, Dublin, CA, USA) in both the optic nerve bending and non-bending eyes before and 1 month after tumor resection (Fig. 7). BCVA was recorded as the decimal visual acuity and converted to logarithm of the minimum angle of resolution (logMAR) notation. The Cirrus HD-OCT ganglion cell analysis (GCA) algorithm automatically segmented the macula into superior, superior nasal, inferior nasal, inferior, inferior temporal, and superior temporal sectors, and measured the GCL + IPL thickness^5^.

### Statistical analyses

Data are presented as the mean ± standard deviation. An unpaired t-test was conducted to compare BCVA and GCL + IPL thickness measurements between the bending and non-bending eyes. The paired t-test was conducted to compare changes in BCVA and GCL + IPL thickness before and 1 month after surgery. The correction between BCVA and ONCBA was examined using Spearman’s correlation coefficient. Statistical significance was set at p < 0.05. Statistical analyses were performed using GraphPad Prism version 6 (GraphPad Software Inc., La Jolla, CA, USA).

## Data Availability

All data generated or analyzed during this study are included in this published article.
